# LobSig is a multigene predictor of outcome in invasive lobular carcinoma

**DOI:** 10.1038/s41523-019-0113-y

**Published:** 2019-06-27

**Authors:** Amy E. McCart Reed, Samir Lal, Jamie R. Kutasovic, Leesa Wockner, Alan Robertson, Xavier M. de Luca, Priyakshi Kalita-de Croft, Andrew J. Dalley, Craig P. Coorey, Luyu Kuo, Kaltin Ferguson, Colleen Niland, Gregory Miller, Julie Johnson, Lynne E. Reid, Renique Males, Jodi M. Saunus, Georgia Chenevix-Trench, Lachlan Coin, Sunil R. Lakhani, Peter T. Simpson

**Affiliations:** 1 UQ Centre for Clinical Research, Faculty of Medicine, The University of Queensland, Herston, QLD 4029 Australia; 2 QIMR Berghofer Medical Research Institute, Herston, Brisbane, QLD 4006 Australia; 3 Institute for Molecular Bioscience, The University of Queensland, St Lucia, Brisbane, QLD 4072 Australia; 4 Pathology Queensland, The Royal Brisbane & Women’s Hospital, Herston, QLD 4029 Australia; 5 Present Address: Pfizer Oncology Research, San Diego, CA 92121 USA

**Keywords:** Cancer genomics, Breast cancer

## Abstract

Invasive lobular carcinoma (ILC) is the most common special type of breast cancer, and is characterized by functional loss of E-cadherin, resulting in cellular adhesion defects. ILC typically present as estrogen receptor positive, grade 2 breast cancers, with a good short-term prognosis. Several large-scale molecular profiling studies have now dissected the unique genomics of ILC. We have undertaken an integrative analysis of gene expression and DNA copy number to identify novel drivers and prognostic biomarkers, using in-house (*n* = 25), METABRIC (*n* = 125) and TCGA (*n* = 146) samples. Using in silico integrative analyses, a 194-gene set was derived that is highly prognostic in ILC (*P* = 1.20 × 10^−5^)—we named this metagene ‘LobSig’. Assessing a 10-year follow-up period, LobSig outperformed the Nottingham Prognostic Index, PAM50 risk-of-recurrence (Prosigna), OncotypeDx, and Genomic Grade Index (MapQuantDx) in a stepwise, multivariate Cox proportional hazards model, particularly in grade 2 ILC cases (*χ*^2^, *P* = 9.0 × 10^−6^), which are difficult to prognosticate clinically. Importantly, LobSig status predicted outcome with 94.6% accuracy amongst cases classified as ‘moderate-risk’ according to Nottingham Prognostic Index in the METABRIC cohort. Network analysis identified few candidate pathways, though genesets related to proliferation were identified, and a LobSig-high phenotype was associated with the TCGA proliferative subtype (*χ*^2^, *P* < 8.86 × 10^−4^). ILC with a poor outcome as predicted by LobSig were enriched with mutations in *ERBB2*, *ERBB3*, *TP53*, *AKT1* and *ROS1*. LobSig has the potential to be a clinically relevant prognostic signature and warrants further development.

## Introduction

Invasive lobular carcinoma (ILC) is the most common ‘special’ type of breast cancer, accounting for 5–15% of all cases. The tumor has distinct morphological and biological features, and clinical behavior compared to the more commonly diagnosed invasive carcinoma-no special type (IC-NST). Typically, ILC tumors display features associated with a good prognosis: lower grade, estrogen/progesterone receptor (ER/PR) positive, HER2 negative and a low proliferative index.^1^ Generally, there is a poorer response to chemotherapy,^2^ yet most patients will respond well to endocrine therapy,^3^ and data from the BIG 1–98 trial suggests that aromatase inhibitors, such as letrozole could be more effective than tamoxifen.^4^ However, ILC has an inherently invasive growth pattern and can be highly metastatic.^5^ Indeed, several large patient cohort studies have demonstrated that the overall long-term outcome for patients diagnosed with ILC may be similar or even worse than it is for patients diagnosed with IC-NST.^3,6^ This presents a conundrum for clinicians with few clues to inform which patients will develop recurrent or metastatic disease.

Loss of the transmembrane cell–cell adhesion molecule, E-cadherin, is a critical molecular event in the natural history of the lobular phenotype. *CDH1* gene mutation, deletion and/or methylation account for the absence of functional E-cadherin complex,^1^ contributing to the lack of cellular cohesion and resulting invasive growth pattern. Many of the clinical challenges associated with diagnosing and managing patients with ILC are directly related to this behavior, including the difficulty in imaging by mammography^7^ and obtaining clear surgical margins. Subsequently, more patients present late, with larger tumors, more frequently involved axillary lymph nodes and requiring higher frequency of mastectomies compared to patients diagnosed with IC-NST.^8^

The genomic profile of ILC has been explored in some depth,^9–11^ revealing that these tumors are more likely to be diploid than IC-NST, and harbor recurrent gains of chromosome 1q, 8q, 16p; deletions of 8p23-p21, 11q14.1-q25, and 16q; and complex, high-level amplifications at 1q32, 8p12, and 11q13.^10–13^ Three large studies have recently presented a more comprehensive examination of the multi-omic landscape of ILC, providing power to tease out alterations enriched in ILC relative to IC-NST.^14–16^ For instance, ILC are typified by *CDH1* and *PTEN* loss, enhanced AKT activation, mutations in *TBX3* and *FOXA1*, and amplification of *ESR1*. Of great interest is the enrichment for potentially actionable mutations in *ERBB2* (HER2, 5.1%) and *ERBB3* (HER3, 3.6%).^14^ Indeed, HER2-negative ILC with high-grade features show an increased frequency of *ERBB2* mutations (15%), especially the pleomorphic variant (26%),^17^ far higher than that reported for breast cancer generally (≤1%, TCGA^18^), but with no significant impact on prognosis.^19^ *ERBB2* mutation in *CDH1*-mutated patients shows a significantly worse outcome than control groups, and indeed in *CDH1*-mutated cancers that have relapsed, there is a high *ERBB2* mutation rate.^20,21^

Analysis of gene expression data has led to the classification of molecular subtypes within ILC.^15,16^ TCGA developed a 60-gene classifier and identified ‘reactive-like’, ‘immune-related’, and ‘proliferative’ subtypes of the disease. The ‘reactive-like’ tumors had enriched stromal/cancer fibroblast signaling and high expression of various myoepithelial genes (including *SOX10*, *KRT14*, *COL17A1*)^15^ and were more likely to also be classified as normal-like using the intrinsic subtyping approach. Whilst this analysis was focused more on biology than prognosis, it was unsurprising that the proliferative group had a worse outcome compared to the immune and reactive-like groups.^15^ Independently, subtyping by a European team also defined an immune-related group with high expression in lymphocyte signaling, together with a hormone-related subgroup with elevated levels of *PGR1*, *ESR1*, and GATA3 protein expression.^16^ However, these two ‘immune’ subtyping approaches do not identify the same cases when applied to the same dataset,^22^ and detailed analyses confirm that ILC broadly have a low level of tumor infiltrating lymphocytes.^22^

Despite clear biological and clinical differences, treatment of IC-NST and ILC remains the same. Prognostication is routinely performed using clinico-pathologic information; namely the Nottingham Prognostic Index (NPI),^23^ which comprises tumor size, grade and lymph node status, and an IHC panel to evaluate ER, PR and HER2 (with or without Ki67, a marker of proliferation).^24^ Ostensibly, the molecular signature market for breast cancer is a busy space (reviewed in ref. ^25^), however the utility in ILC of some of the existing commercial tests remains to be seen, and uptake is by no means global. While most focus on ER+ tumors, notably, none of these signatures account for tumor morphology in their algorithms. The Genomic Grade Index (GGI/MapQuantDx™) panel has been shown to be more powerful than grade alone in the ILC population,^26^ while MammaPrint® has validated value only in node negative ILC patients.^27^ The clinical utility in ILC of the 21-gene signature, OncotypeDx®, remains unclear with two studies showing classification of 42%^28^ and 35.5%^29^ of patients as being as of intermediate risk (IR; managing the IR designated patient is clinically challenging^30^) and further studies indicate limited additional value over histology.^31^ Prosigna® is the commercial diagnostic test based on the PAM50 ‘intrinsic’ subtyping. It generates a Risk of Recurrence score (ROR) and has a better prognostic value than that of the OncotypeDx test, in ER+ node negative patients.^32^ Again, the utility of Prosigna® in ILC specifically is unknown, and a recent study on its utility in breast histological special types excluded ILC.^33^ A recently reported five-transcript metagene, EarlyR, has shown prognostic power for recurrence-free survival over 8 years in ER+ tumors, however there is no discussion of histology.^34^

Here, we present an integration of gene expression and copy number data to identify genes influencing ILC behavior and prognosis. Through this combination of approaches we have developed a 194 metagene signature, which we have termed LobSig, that could add significant prognostic power to the standard clinical information for patients with ILC.

## Results

### Genomic features of ILC associated with outcome

**Fig. 1:**
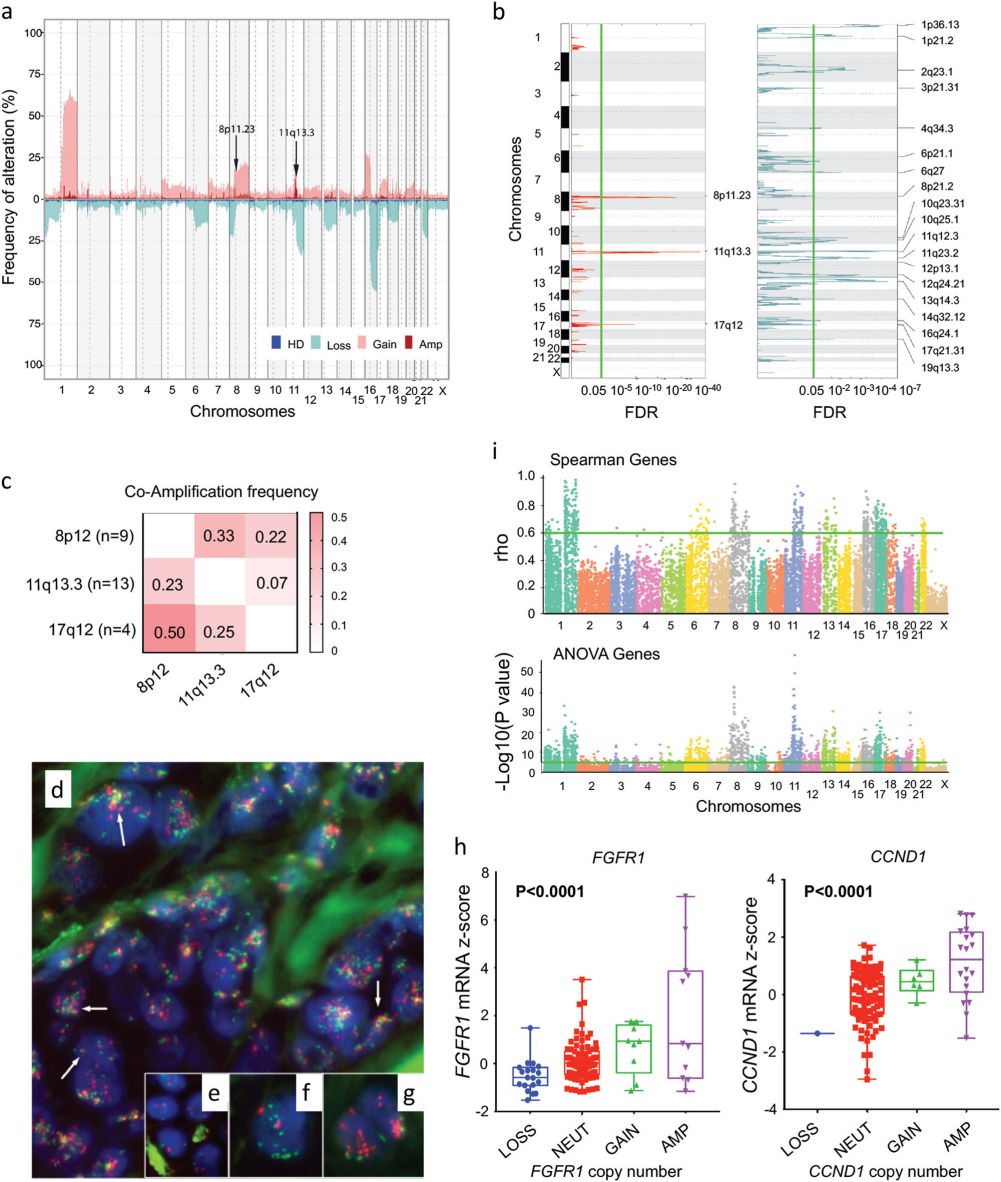
ILC genomic landscape. **a** Copy number landscape of 303 ILC tumors as demonstrated by frequency of alteration (%, *Y*-axis) across the genome (chromosomes on *X*-axis). Red, amplification (Amp); pink, Gain; light blue, Loss; dark blue, homozygous deletion (HD). **b** GISTIC significant focal alterations. Amplifications in red and deletions in blue, significant false discovery rate (FDR) in green. **c** Heatmap of frequency of recurrent co-amplifications in ILC tumors. **d** FISH analysis showing co-amplification (yellow) of *FGFR1* (green) and *CCND1* (red) in an ILC case identified as having co-amplification of 8p12 and 11q13 by SNP array. Note increased numbers of signals for both genes in individual nuclei; signals also often clustered/joined (arrows) suggesting a complex clustered rearrangement process involving translocation between these gene regions. **e** shows normal cells diploid for both genes; **f** shows a tumor cell nucleus with multiple copies of *FGFR1* (green) and a chromosome 8 centromere probe (red); **g** shows two tumor nuclei analyzed for *CCND1* (red) and a chromosome 11 centromere probe (green). LCIS present in the same section displayed the same pattern of co-amplification (not shown), while no evidence of gene copy number change was seen in surrounding columnar cell lesions (not shown). **h** Boxplot of copy number versus mRNA expression *z*-scores of FISH targets *FGFR1* and *CCND1*; central line is median, with whiskers extending from the 25th and 75th percentiles. **I** Spearman genes plotted as *ρ* across chromosomal location (*X*-axis) and ANOVA genes plotted as −log *P* value across chromosomes. Green lines represent cut-off point of significance (*ρ* > 0.06; *P* < 0.00001)

Several studies have reported the DNA copy number landscape of ILC.^10–13^ Here, single nucleotide polymorphism (SNP) array data from three cohorts was merged to review this landscape (Fig. 1a) in a large series of cases at higher resolution (*n* = 303; Fig. 1a; Supplementary Fig. 1 for individual cohort data). Previously defined recurrent alterations were identified in this pooled ILC cohort, with large chromosome level gains seen on 1q, 8q, 11q and 16p; and deletions on 1p, 6q, 8p23-p21, 11q14.1-q25, 13q, 16q and 22. Recurrent, high-level amplifications were also identified 8p12-p11.2 (7%), 11q13.3 (12%) and 17q12 (2%; Fig. 1b) and significant focal deletions were defined at various loci, including 1p, 11q and 13q (Fig. 1b; Supplementary Table 2). GISTIC analysis of the TCGA cohort, identified putative drivers in these regions including *CCND1* and *ORAOV1* (11q13.3), *FGFR1* and *LETM1* (8p12), and *ERBB2* (17q12) (Supplementary Table 2). GISTIC focal alterations were then associated with breast cancer-specific survival (BCSS) data to identify regions that are highly prognostic in ILC tumors (Supplementary Table 3). Key prognostic regions of deletion as assessed by Logrank include 19p13.3 (*P* = 0.0031); 2q23.1 (*P* = 0.0034); 8p21.2 (*P* = 0.0036); 14q32.12 (*P* = 0.0192); and 1p21.2 (*P* = 0.0218) (Supplementary Fig. 2). A poorer prognosis is associated with the presence of amplifications in any of the following three regions, or combinations of, 11q13.3, 8p11.23, and 17q12 (*P* = 0.0383) (Supplementary Fig. 3).

Interestingly, of the nine ILC tumors with amplification at 8p12-11.2, three (33%) had co-amplification with 11q13-q14.1, as previously reported (Fig. 1c).^11,12,35^ This event was shown recently to be a co-evolution, and likely an early, critical event in tumorigenesis.^35^ FISH analysis using gene-specific probes for *FGFR1* (8p11) and *CCND1* (11q13.3) (GISTIC-identified putative driver genes Supplementary Table 2; Fig. 1h), confirmed this co-amplification event in a tumor from the UQCCR cohort, including in an adjacent component of Lobular Carcinoma in situ (LCIS; Fig. 1d–g). All tumor cells harbored multiple signals for each gene and co-clustering of signals indicating that this was part of a complex structural rearrangement and amplification event,^35^ and was likely to be an early and critical driver alteration in the evolution of some tumors.

### Gene expression characteristics associated with outcome in ILC

ILC cases from the METABRIC cohort, with both gene expression and clinical follow up data, were interrogated to determine if gene expression changes were associated with patient survival (*n* = 101; Supplementary Fig. 4). A supervised analysis of differential gene expression profiling of ‘good‘, and ‘poor’ BCSS outcome groups identified a total of 856 probes/772 genes (Supplementary Table 4). Chi-squared analysis revealed that the two sample subgroups were significantly associated with PR status (*P* = 9.541e–05), PAM50 subtype (*P* = 0.0005) and outcome (*P* = 4.583e–05) (Supplementary Table 5); gene cluster 1 was enriched for good outcome, normal-like/luminal A, PR positivity and grade 2 tumors while gene cluster 2 was enriched for poor outcome, Luminal B/HER2/Basal, PR negativity, and grade 3 tumors. This panel of genes were analyzed using GeneGo Pathways Software (MetaCore) to identify pathways/functional modules that might be driving the behavior of these subgroups (Supplementary Table 6). The poor outcome cluster showed significant enrichment of the ‘cell cycle initiation of mitosis’ module (FDR = 6.788e–06) and also of the ‘progesterone-mediated maturation’ module (FDR = 9.165e–06). The modules down-regulated in the poor outcome cluster include ‘Gonadotropin-releasing hormone signaling’ (FDR = 0.00004), ‘YAP/TAZ co-regulation of transcription’ (FDR = 0.002) and various immune-signaling pathways, such as ‘IL18 signaling’ (FDR = 0.002).

### Identifying copy number-driven expression changes

In order to identify copy number-driven expression changes, we integrated gene expression and copy number data using two complementary approaches: Spearman rank order and ANOVA meta-analysis. The rationale for this approach is detailed in Supplementary Fig. 4. This analysis was performed in all three datasets independently, before combining the data in a meta-analysis using either a Dersimonian Laird (for the Spearman generated data) or Stouffers *Z*-score (ANOVA data) method. A total of 1896 genes were identified from the ANOVA analysis (*P* < 0.00001) and 428 genes from the Spearman analysis (combined effect size >0.6); 1501 genes were unique to the ANOVA analysis (Supplementary Table 7) and 33 genes were unique to the Spearman analysis (Supplementary Table 8); 395 genes were common between both methods (Supplementary Table 9). Many of the genes were present in regions of the genome with recurrent alterations (1q, 8p, 8q and 11q, 13q, 16p and 16q), most notably from regions of high-level amplification at 8p12-11 and 11q13-14 (Fig. 1i; Supplementary Tables 7–9). Some of the top genes identified from the ANOVA analysis include those at 11q13 including *INTS4* (*P* = 5.957e−59), *CLNS1A* (*P* = 4.110e–50), *FADD* (*P* = 6.441e–42), *PRKRIR* (*P* = 4.247e–39) and *CTTN* (*P* = 6.237e–39); and at 8p12, *ASH2L* (*P* = 2.777e–43), *PROSC* (P = 1.0348e–42), *BRF2* (*P* = 1.057e–40) and *LSM1* (*P* = 6.072e–40 (Supplementary Table 7; Supplementary Fig. 5). The top genes from the Spearman analysis were enriched at the 1q locus, specifically *GNPAT* (*ρ* = 0.969), *VPS45* (*ρ* = *0*.*959*), *PRCC* (*ρ* = *0*.*948*), *COG2* (*ρ* = 0.946) and *ARV1* (*ρ* = 0.928) and at 8p12, e.g. *LSM1* (*ρ* = *0*.*936*) (Supplementary Table 8; Supplementary Fig. 6).

### Synergizing prognostic capabilities

**Fig. 2:**
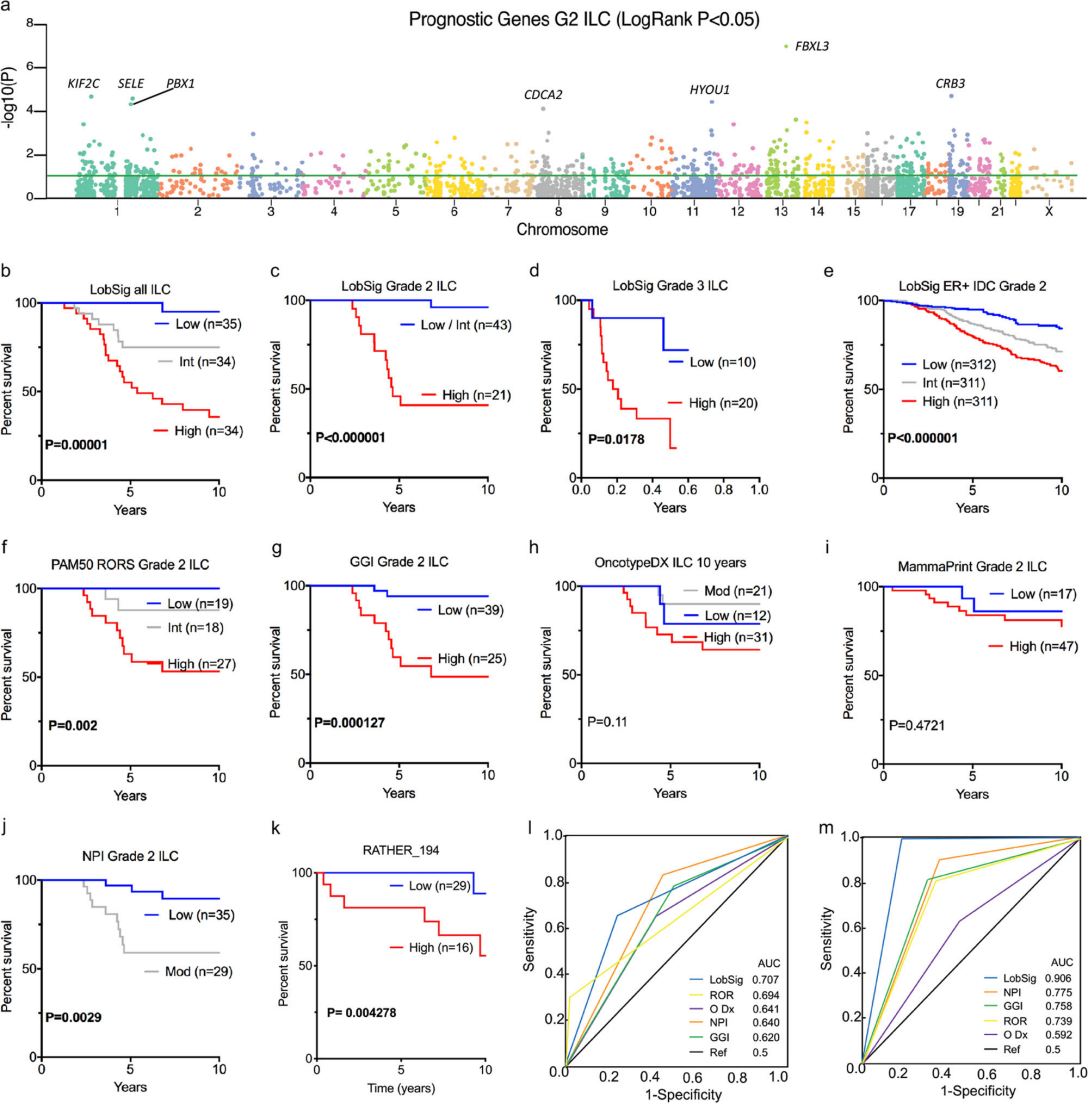
LobSig is an excellent prognostic tool with superior performance in ILC tumors. **a** Manhattan plot of the prognostic grade 2 ILC genes across all chromosomes; with logrank *P* < 0.05 marked as green line. Kaplan–Meier curves of signature stratified populations that retained an independent prognostic role for BCSS. LobSig in all ILC **b**; grade 2 only **c**; grade 3 only **d**; Grade 2, ER-positive IC-NST **e**. Considering the population of grade 2 ILC only, existing signatures stratify as follows: **f** PAM50 RORS, **g** GGI, **h** OncotypeDX, **i** MammaPrint, and **j** NPI. **k** is the LobSig194 stratification seen in cases unique to the RATHER cohort. *P*-value is logrank. Blue lines indicate low-risk patients, gray indicates patients with intermediate-risk and red lines indicate high-risk patients. Receiver operator characteristic (ROC) curves comparing performances of prognostic gene signatures in ILC tumors **l** and Grade 2 ILC tumors **m**

Each gene from the integrative and GEX analyses was evaluated independently to identify the association with outcome to ensure robust prediction in the resulting lobular-specific meta-gene. Survival associations were assessed in all ILC tumors and then exclusively in grade 2 ILC to account for the disproportionately high number of grade 3 ILC in the METABRIC cohort. Filtering was dependent on stringent requirements: (i) logical, monotonic spread of the tertile-split KM curves of mRNA expression; (ii) consistency between multiple probes for the same gene; and (iii) significant separation of the curves based on the logrank test, as plotted in Fig. 2a (Supplementary Table 10). A 194 gene set, which we termed ‘LobSig’, comprises the resultant collection of prognostic genes. The gene set shows limited similarity with many of the available commercial signatures (Supplementary Table 11). Comparing LobSig with OncotypeDx,^36^ TCGA 60 gene classifier;^15^ RbSig^37^ GGI;^38^ MammaPrint;^39^ PAM50;^40^ EarlyR;^34^ ER and CIN attractors;^41^ proliferation_AURKA,^42^ 176/194 genes are unique to LobSig. The LobSig genes most commonly encountered across the various metagenes analyzed were *BIRC5* and *CCNB1*, present together in 6/10 tested signatures. LobSig contains 27 genes (14%) considered to be cell-cycle/proliferation-related (cf. GGI (54%) RbSig (74%)). *SFRP1* is the sole gene in common between LobSig and the TCGA 60 gene classifier^15^ and its loss correlates with poor overall survival in breast cancer patients.^43^

### LobSig outperforms existing signatures in prognostication in silico

**Fig. 3:**
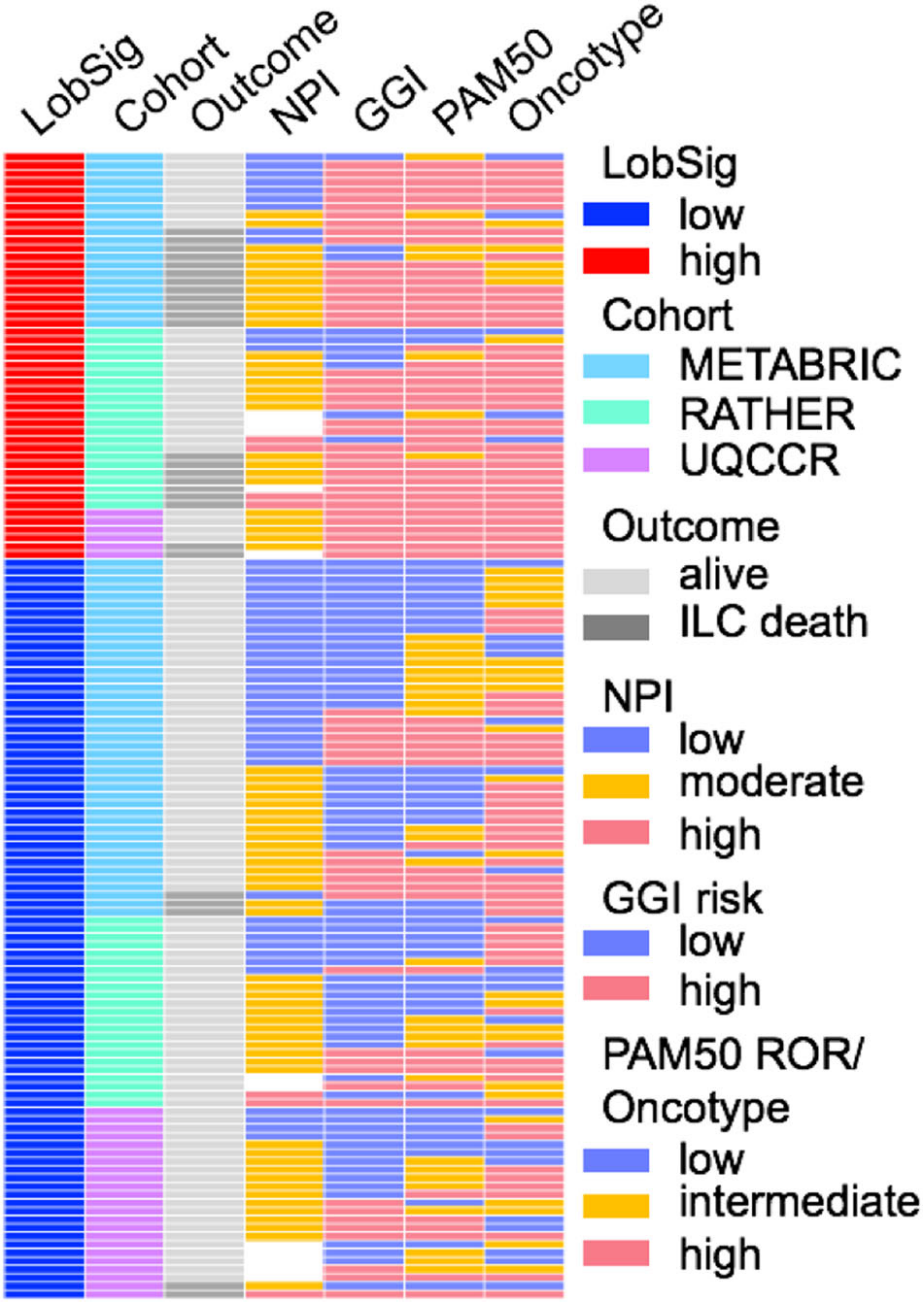
LobSig does not simply recapitulate existing risk evaluating signatures. Case by case assessment of risk predictions. Comparison of risk scores of 138 cases of grade 2 ILC. NPI Nottingham Prognostic Index, GGI Genomic Grade Index, ROR risk of recurrence

LobSig is highly prognostic in unselected ILC, and specifically in grade 2 and grade 3 ILC tumors, as well as to a lesser degree in ER-positive, grade 2 IC-NST cases (Fig. 2b–e). LobSig stratifies ILC significantly compared to existing signatures (Fig. 2f–i) while neither OncotypeDX nor MammaPrint are prognostic exclusively in this tumor type (Fig. 2h, i). LobSig outperforms existing signatures in both a univariate (*P* = 9.0 × 10^−6^) and multivariate context (*P* = 3.14 × 10^−4^; Supplementary Tables 12–14), and shows greater prognostic capability than the NPI (Fig. 2j). Considering the discovery cohorts separately, LobSig stratifies 17.3% grade 2 ILC in TCGA and 30% grade 2 ILC in METABRIC as LobSig high, with an increased risk of a poor outcome (Supplementary Fig. 8). Furthermore, using the RATHER cohort as an independent validation set, 31.4% are stratified as LobSig high (Fig. 2k; Supplementary Fig. 7). LobSig is particularly effective in grade 2 ILC tumors (AUC = 0.906, Fig. 2m), versus all ILC (AUC = 0.707; Fig. 2l). Figure 3 demonstrates the case-by-case data of LobSig risk compared to the risk scores generated by NPI, GGI, PAM50 ROR and OncotypeDx; the heterogeneity of the alternative risk scores within the LobSig groups confirms that LobSig does not simply recapitulate the risk scores of existing signatures.

**Fig. 4:**
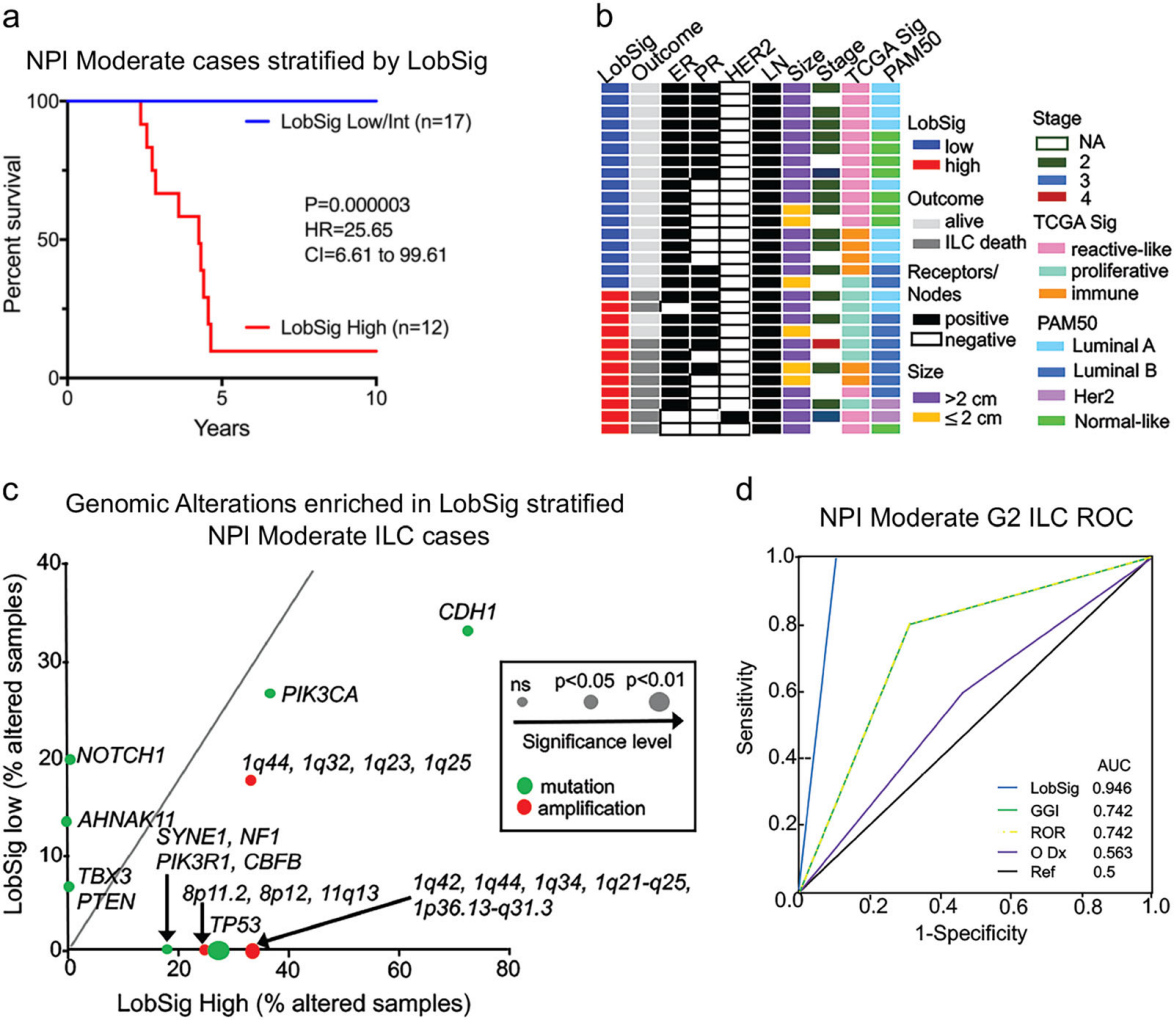
LobSig adds prognostic value to NPI. **a** BCSS of the LobSig-stratified NPI moderate grade 2 ILC population. **b** Heatmap showing the clinical and molecular characteristics of NPI moderate LobSig-stratified tumors with blue corresponding to LobSig low, and red corresponding to LobSig high. **c** Scatterplot showing the enrichment of gene-centric mutations and focal copy number alterations in the LobSig-stratified NPI moderate cohort. Significance represented by dot size; mutations in green, amplifications in red. **d** Receiver operator characteristic (ROC) curve comparing performances of different prognostic gene signatures in the NPI moderate grade 2 ILC cohort. AUC area under curve, Ref reference, O Dx OncotypeDx

Of the 126 cases assigned an NPI risk category, 49 (38.9%) were good, 7 (5.6%) were poor but 70 (55.5%) were assigned a moderate risk. Focusing on NPI moderate cases (grade 2; METABRIC, *n* = 29), stratification with LobSig was performed to determine whether LobSig would add value, and be able to re-assign the ‘moderate’ cases. Figure 4a shows that LobSig is highly prognostic in the NPI moderate grade 2 tumors within the cohort. Interestingly, there is no clear difference between the groups in terms of histopathological characteristics (Fig. 4b). Unique molecular subgroups were prevalent among LobSig-stratified tumors (Supplementary Table 15; Fig. 4b) with enrichment for Luminal B and TCGA proliferative type in the LobSig high group, and Luminal A/normal-like and TCGA reactive-like in the LobSig low group (Fig. 4b). There was a significant enrichment of *TP53* mutation in the LobSig high group, consistent with a poor outcome tumor type (Fig. 4c). LobSig is the most accurate of the signatures tested in predicting survival outcomes for grade 2 NPI moderate cases (Fig. 4d).

**Fig. 5:**
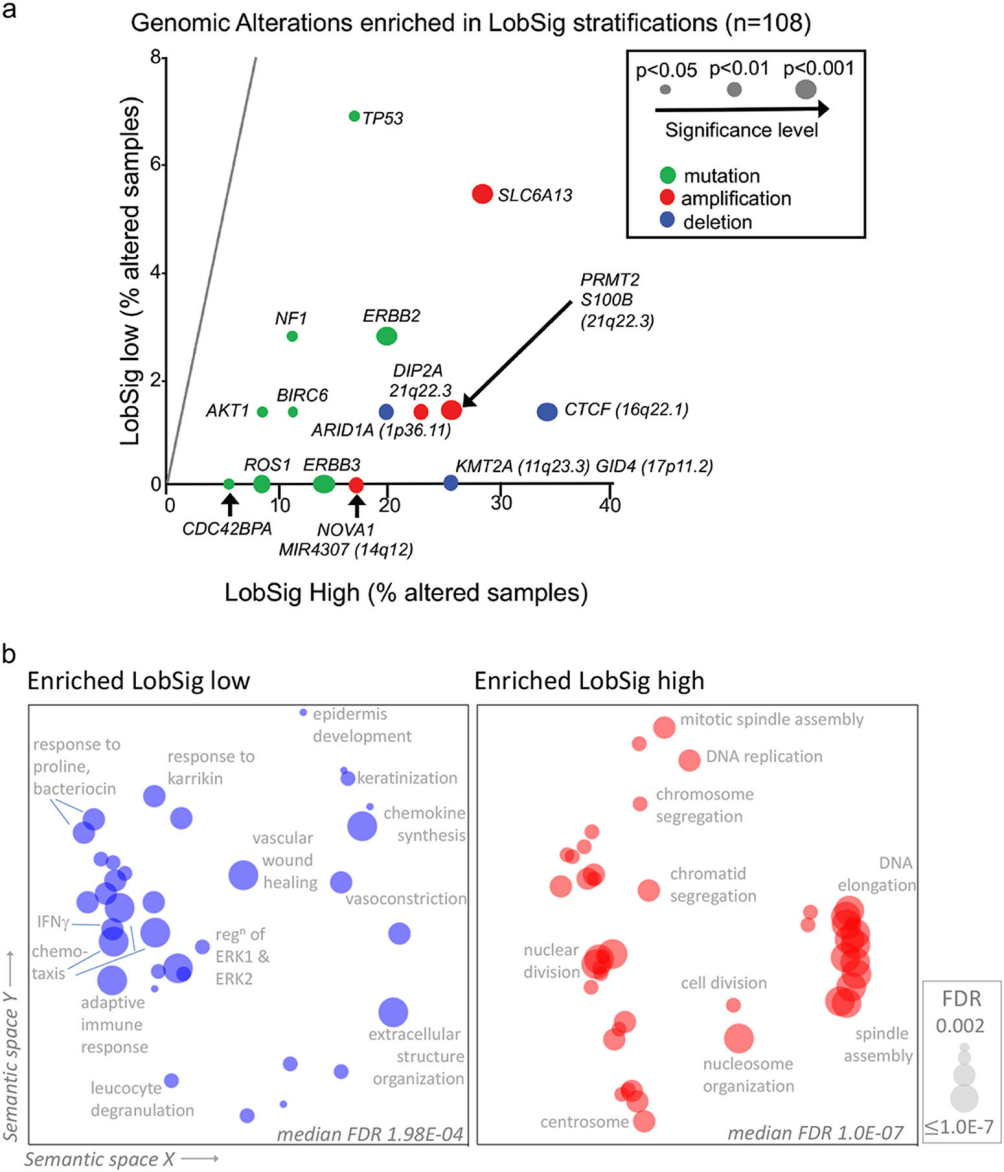
Features of LobSig-stratified tumors. **a** Genetic landscape shown in scatterplot of gene-centric mutations and driver alterations that characterize the LobSig high-risk group. Samples analyzed were grade 2 ILC tumors in the METABRIC and RATHER cohorts (*n* = 108). Significance represented by dot size; mutations in green, amplifications in red, deletions in blue. **b** Gene Ontology analysis of the genes differential expression between LobSig high and low tumors is visualized with REVIGO, where the *X* and *Y* distance means similar terms are closer together. The terms generated using the METABRIC and TCGA cohorts were combined, and only those terms with a false discovery rate (FDR) of *q* < 0.002 were included in the REVIGO analysis. Note that the FDR is represented by size. Reg^n^ regulation

To identify genetic features discriminating the LobSig stratification, an assessment of genetic alterations and their enrichment was made (Fig. 5a, Supplementary Table 15). This analysis showed LobSig high tumors were enriched for mutations in *ERBB3* (*P* = 0.00007), *ERBB2* (*P* = 0.0002), *BIRC6* (*P* = 0.005), *AKT1* mutations (*P* = 0.02), *ROS1* (*P* < 0.01); amplifications of *PRMT2* (*P* = 7.329e–08), *S100B* (*P* = 7.33e–08) and *DIP2A* (*P* = 7.99e–07; 21q22.3); and for deletions of *CTCF* (16q22.1; *P* = 8.41e–11), *C17ORF39* (17p11.2; *P* = 4.597e–09) and *ARID1A* (1p36.11; *P* = 8.045e–06). The LobSig low tumors showed a relatively quiet genome.

In order to define broader molecular differences between LobSig low and high tumors, Gene Ontology (GO) terms were assigned to the differentially expressed genes, revealing enrichment of several pathways (FWER *P*-value < 0.05; Supplementary Tables 16 and 17). These terms are visualized using REVIGO (Fig. 5b), which summarizes the semantic similarity of the GO terms. The pathways upregulated in LobSig high tumors were cell cycle processes including DNA replication, chromosomal segregation, mitotic nuclear division, organelle fission, mitotic spindle organization. Pathways enriched in the LobSig low group were diverse and included various immune pathways, such as regulation of leukocyte chemotaxis, monocyte chemotaxis, chemokine-mediated signaling and adaptive immune response.

## Discussion

Despite clear biological and clinical differences, treatment of IC-NST and ILC remains the same. It is currently impossible to predict ILC clinical course at diagnosis, as a result of homogeneity in the standard diagnostic criteria for ILC. Molecular diagnostic tests, such as OncotypeDx, remain of limited value for ILC, since there is a paucity of data on their suitability and they were not developed on this tumor histology. Differentiating which patients will do well long-term on endocrine therapy and could be spared chemotherapy treatment-associated morbidity, and, which patients require aggressive treatment remains unclear. In this study, we have derived the first meta-gene signature focused on prognostication in ILC. LobSig results from the integrated curation of transcripts and genomic regions, in the context of breast cancer-specific survival. Transcripts from previously identified regions of interest^10–13^ in the lobular genome are well represented in the signature. The meta-gene is remarkably robust, out-performing existing signatures in the prognostication of ILC patient outcome. As expected, given our lobular-centric rationale, there are limited similarities with existing signatures, further supporting that some of these genes are unique to the ILC biology. A component of the gene set relates to proliferation, however, this is unlikely to be the lone driving force of LobSig’s prognostic power, given its improvements in stratification of risk over grade and other signatures. Naturally, there are limitations associated with an in silico study predicting the prognosis of ILC patients: their often long time to relapse makes finding extensive cohorts with molecular profiling data for both discovery and validation challenging. We present a gene set derived acknowledging these limitations, but with the capacity to be refined and developed to the benefit of ILC patients in the future. In addition, LobSig provides a detailed examination of the molecular variability in an otherwise clinicopathologically homogeneous cohort.

Stratification with LobSig identified a group of low-risk tumors, which showed an enrichment for luminal A phenotype, immune-related pathways, and the TCGA immune-enriched subtype. The LobSig low samples have the best BCSS outcomes, and the impact of their immune-enrichment appears similar to that of triple-negative breast cancer, whereby higher levels of tumor infiltrating lymphocytes correlated with a better prognosis.^44^ There are few published datasets of ILC with detailed TILs analysis, however Desmedt et al.^22^ show in a comparative analysis that ILC generally have low levels of TILs compared to IDC. They also found, somewhat paradoxically, that those ILC patients with high TILs were of young age, with proliferative, LN+ tumors.^22^ However, immune-enriched ILC from TCGA had a better outcome than those designated proliferative.^15^

LobSig high tumors were enriched for the Luminal B subtype, an expected finding independently confirming previous data that luminal B ILCs have a poorer outcome than luminal A ILC.^45^ Luminal B tumors are known to have a higher proliferation index with higher expression of *CCNB1*, *MKI67* and *MYBL2* compared to Luminal A tumors.^46^ Similarly, a subset of LobSig high tumors are also classed as the TGCA proliferation subtype; however, only 1 gene is shared by both LobSig and the TCGA 60-gene classifier (*SFRP1*). We found no correlation between *MKI67* expression and the LobSig high group of tumors, and only 14% of LobSig gene set annotated for proliferation. A surprising finding is that *MYC* expression is low in the LobSig high cohort. MYC is recurrently altered across ILC and a common driver of tumor progression and recurrence in ER-positive breast cancers generally, however this may not be the case in LobSig high tumors.^47,48^ The signature captures a biology driven by the combination of multiple different genomic alterations (amplifications of 1q, 8p, 11q, 17q12; mutations in *TP53*, *ERBB2/3*; losses of 13q). All these events occurred at relatively low frequencies but collectively, they drive this apparent ‘aggressive’ behavior. Ki67 is unlikely to be sufficient to capture all this diversity, however, the GGI is good at capturing a similar biology. PAM50 highlights some enrichments of intrinsic type in the LobSig High group (e.g. HER2, luminal B), while OncotypeDx was not prognostic in this dataset. In fact, several papers have pointed to the limited value in ILC patients, with recent SEER dataset analyses showing that OncotypeDx offers little value above standard histopathology in ILC and other low-risk subtypes;^31,49^ many recent studies concede that the relevance of OncotypeDx ILC requires further study.^28,29,50^ Overall, LobSig appears to have increased value than existing signatures in the lobular context.

There was a notable prevalence of *ERBB2* (20%), *ERBB3* (14.28%), *AKT1* (8.57%) and *ROS1* (8.57%) mutations in the LobSig high group, raising exciting possibilities for applying targeted therapies in LobSig high tumors, with evidence emerging of the value of anti-HER2 therapies,^19,51–54^ AKT inhibitors^55^ and the recently described ROS1 inhibitors via synthetic lethal interaction with *CDH1* mutant ILC.^56^ Multivariate analysis demonstrated the significant value of LobSig above individual clinico-pathology features, but more importantly, the value of this signature resides in its ability to stratify the NPI moderate tumors—effectively moving from the ‘intermediate’, unclear group, into one of two groups with clear prognostic outcomes. The data presented here supports that LobSig low-risk patients need not receive adjuvant chemotherapy. Our signature is not predictive for chemotherapy administration per se, but likely identifies a group of ILC patients in whom chemotherapies may be beneficial. A paucity of highly annotated ILC cohorts with sufficient follow-up, as well as molecular profiling data in a clinical trial setting, precludes us from determining if and whether there are specific therapies that may have efficacy.

In conclusion, we present the molecular signature, LobSig, which captures the peculiar genomic landscape of ILC tumors, and together with clinico-pathology information, provides a robust mechanism for prognostication in ILC. This signature warrants further analysis and development, and validation on expanded retrospective cohorts of ILC with detailed treatment information.

## Methods

### Sample cohort details

Fresh frozen tumors and matching blood samples were accessed from the Brisbane Breast Bank (BBB) at the University of Queensland Centre for Clinical Research (UQCCR) and from the Australian Breast Cancer Tissue Bank (ABCTB) based at the Westmead Institute for Medical Research. These cases constituted the in-house ‘UQCCR’ cohort. All patients provided written, informed consent to the use of their tissues for research and the study had ethics approval from the Human Research Ethics Committee (The University of Queensland (2005000785) and Royal Brisbane and Women’s Hospital (2005/022)).

DNA and RNA were extracted from frozen tissue sections by either collecting frozen sections directly into extraction tubes or following needle dissection to enrich for tumor cellularity. Tumor cellularity was estimated by a pathologist (G.M., S.R.L.) from adjacent-stained frozen sections, with samples requiring 70% observed tumor cellularity to progress (thus reducing the available samples for analysis significantly). QIAgen extraction kits were used (QIAgen, Chadstone, Vic, Australia). Quantification and quality assessment of nucleic acids were performed using the Qubit dsDNA BR and RNA BR assays (Invitrogen, Scoresby, Australia) and Bioanalyser RNA 6000 Nano assay (Agilent, Mulgrave, Vic, Australia).

### Published ILC datasets

Molecular profiling data on ILC tumors was accessed from a number of published studies. In brief: discovery set comprised *n* = 146 ILC from The Cancer Genome Atlas (TCGA) data portal (http://cancergenome.nih.gov/, data status as at May 15, 2014);^15^ *n* = 125 ILC from the METABRIC cohort (EGAS00000000083)^57^ (excluding those classified as low cellularity or not annotated for cellularity); validation set, *n* = 45 unique ILC cases from the RATHER cohort (GSE68057).^16^ Supplementary Table 1 describes the clinicopathological features of these cohorts and the context in which they were used in this study. Gene expression data for ILC samples from TCGA were obtained as raw RNA-Seq counts for each gene. This data was voom transformed^58^ using the limma package^59^ in preparation for integration with DNA copy number data (see below).

### Gene expression profiling and analysis

Gene expression profiling of UQCCR samples was performed using the Whole-Genome Gene Expression Direct Hybridization Assay (Illumina, Scoresby, VIC, Australia) as per protocol. Briefly, the Epicentre TargeTAmp kit (Illumina) was used to label 250 ng of RNA, and the samples were hybridized to the HT12 v4 chips before scanning on the Illumina iScan. Data was analyzed using arrayQualityMetrics^60^ in BioConductor and is available from GEO (GSE98528).

Following quantile normalization within each cohort (UQCCR and METABRIC), the datasets were merged and batch effects were removed using ComBat.^61^ Prior to hierarchical clustering, the gene expression data was standardized through median-centering and dividing by median absolute deviation normalized expression changes. Samples were then correlated using Spearman distance, and genes were correlated using Pearson distance. Gene expression data from UQCCR cohort (*n* = 25) and RATHER (*n* = 45) was normalized (quantile and Robust Multichip Average (RMA), respectively) separately and subject to the PAM50 classification using the bioclassifier R scripts.^62^ The distance to each centroid was calculated using Spearman rank correlation. The centroid with the largest positive correlation was assigned as the subtype of each sample.

### DNA copy number profiling and analysis

Tumor DNA (200 ng) and matched normal DNA from UQCCR cases was profiled using Illumina Omni2.5-8, V1.1 SNP arrays, according to manufacturer’s instructions. Tumor cellularity was measured with *qPure*.^63^ Copy number was quantified and summarized using *GAP*^64^ and *GISTIC 2*.*0*.^65^ *GISTIC 2*.*0* parameters for significant deletions and amplifications were set at 95% confidence, with a *q*-value of <0.25 deemed significant. Frequency plots were generated as detailed in the Supplementary Information. Discrete GISTIC focal copy number alterations were obtained from the METABRIC cohort. Only those samples that were grade 2 and had available survival data were kept for further analysis (*n* = 63). The *rms* package was used to associate the CN events with outcome. Associations of copy number events and clinical pathological features were performed using a chi-squared analysis.

### Fluorescence in situ hybridization (FISH)

FISH was performed using probes specifically targeted to genes *FGFR1* and *CCND1* (Empire Genomics, Buffalo, NY, USA), labeled with 5-Fluorescein dUTP and 5-carboxyl-x-rhodamin dUTP, respectively. Four micron tissue sections were treated using the SPOT-Light Tissue Pretreatment Kit (Life Technologies), with heat pre-treatment performed for 40 min at 99 °C and enzyme digestion performed for 5 min at 37 °C. The slides were then dehydrated and the probe applied as per manufacturer’s instructions (1:4 dilution Empire Genomics). Denaturation was performed for 3 min at 83 °C and hybridized overnight at 37 °C in a humidified chamber. Slides were mounted and counterstained using ProLong Diamond anti-fade with DAPI (Life Technologies). DNA copy number was assessed by scoring the number of signals seen in at least 20 discrete tumor cell nuclei within each high-power field.

### Frequency plots

Genome-wide frequency plots for somatic CNAs (from UQCCR, TCGA, and METABRIC cohorts) were generated using the *copynumber* package in R.^66^ For each cohort either absolute copy number values (UQCCR cohort *n* = 30), binned copy number states (METABRIC cohort *n* = 125), or CBS-smoothed log ratios (TCGA cohort *n* = 146) were available and distinct thresholds were determined for each cohort. For the UQCCR cohort, thresholds for calling copy changes were: gain, between 2 and 5 copies of the region; loss, <2 copies; amplification, >6 copies; or homozygous deletion, 0 copies. For TCGA data the thresholds for calling copy gains (CBS smoothed log ratios >0.3), losses (CBS smoothed log ratios <−0.3) and amplifications/homozygous deletions, and sample-specific thresholds from GISTIC were used. The copy number states (i.e. GAIN, LOSS, AMP (amplification), HOMD (homozygous deletion)) were predefined for METABRIC samples.

### Integration of DNA copy number of gene expression data

In order to identify genes that were altered by copy number we searched for segments that overlap with whole gene annotations from Ref Seq (hg19 and hg18 respectively). This was necessary as the segmented data from TCGA was aligned to hg19 while the METABRIC-segmented data was aligned to hg18. Two sample by gene copy number matrices were generated: one matrix was a continuous log-ratio matrix (CBS) smoothed, while the second matrix was the discrete absolute copy number state. For each sample (s) and each gene (g), genes that fell completely within a CBS-derived segment were retained, and assigned that copy number alteration state and corresponding log-ratio C(s,g),L(s,g), respectively. If a transcript of a gene was broken by a set of segments then the C(s,g),L(s,g) was assigned based on the maximal severity based on a relationship denoted below.

$${\mathrm{C}}\left( {{\mathrm{s}},{\mathrm{g}}} \right) = {\mathrm{CNstate}}\left( {{\mathrm{argmax}}\left( {{\mathrm{absolute}}\left( {{\mathrm{severity}}\left( {{\mathrm{CNstates}}} \right)} \right)} \right)} \right.$$

$${\mathrm{L}}\left( {{\mathrm{s}},{\mathrm{g}}} \right) = {\mathrm{LogR}}\left( {{\mathrm{argmax}}\left( {{\mathrm{absolute}}\left( {{\mathrm{severity}}\left( {{\mathrm{CNstates}}} \right)} \right)} \right)} \right.$$

$${\mathrm{Severity}} = \left\{ ({\ NEUT\ ,0),} \right.$$

$$(\ {\mathrm{HOMD}}\ , - 2),(\ {\mathrm{AMP}}\ ,2),$$

$$\left. {(\ {\mathrm{HETD}}\ , - 1),(\ {\mathrm{GAIN}}\ ,1)} \right\}$$

Spearman correlation and ANOVA were applied to integrate gene copy number and expression level from the UQCCR, TCGA, and METABRIC data sets. Spearman correlation was performed using the log-transformed gene expression values and CBS-smoothed log ratios; genes with Spearman rho (*ρ*) ≥ 0.6 were retained for further analysis. A meta-analysis of Spearman correlations was performed using a random effects model (Dersimonian and Laird method^67^) weighting each study by the inverse variance using the metacor() function in the *meta* package.^68^ Genes with a combined effect size value >0.6 were retained. An ANOVA was also performed, testing the relationship between gene dosage and expression level. *P*-values from each dataset were corrected for multiple hypotheses testing using the Benjamini–Hochberg method. We then performed a meta-analysis of the ANOVA analysis using Stouffer’s *Z*-score weighting each study by the sample size. Genes with a combined *P*-value < 0.00001 were retained.

### LobSig development

#### Score calculation

Each gene was assigned a coefficient of either 1 or −1 based on whether high or low expression was associated with poor outcome, respectively. Scores were assigned to each sample in the cohort using sig.score() in the *genefu* package.^69^ This approach uses the linear combination of gene expression values calculating the mean expression of the positive probes subtracting the calculated mean expression of negative probes and standardizing both measurements by the fraction of positive and negative probes in the signature.

$${\mathrm {Score}}\,{\mathrm {per}}\,{\mathrm {sample}} = \left( {\frac{{\mathop {\sum }\nolimits_{t = 0}^{Np} {\mathrm {Expression}}}}{{Np}}} \right) \ast \frac{{Np}}{N} - \left( {\frac{{\mathop {\sum }\nolimits_{t = 0}^{Nn} {\mathrm {Expression}}}}{{Nn}}} \right) \ast \frac{{Nn}}{N}$$

where *N* is the total number of probes in the signature, *Np* is the total number of positive probes in the signature and *Nn* is the total number of negative probes in the signature.

In order to increase the dynamic range of the LobSig scores, the gene expression data for each sample was rescaled, so the expression value was between 0 and 100, based on the following equation:

$${\mathrm{rescaled}}\,A = \frac{{A - \min \left( A \right)}}{{\max \left( A \right) - \min \left( A \right)}} \times 100$$

where *A* is the expression values of LobSig genes in a sample and rescaled *A* is the rescaled expression values of LobSig genes in a sample.

#### Model training

The cohort with computed LobSig scores were split into five separate folds for five-fold cross-validation using the createFolds function in the R package *caret* (https://www.jstatsoft.org/article/view/v028i05), which involves splitting the dataset into five folds and selecting an optimal cutoff for prediction of deceased cases with LobSig score as a predictor from the four folds. For each iteration, the optimal cut off was selected by ROC (i.e. maximize the sum of sensitivity and specificity) using the R package *ROCR*.^70^ The optimal cutoff was used to classify the remaining samples in the fifth fold as either LobSig High or LobSig Low; this process is repeated until all of the samples were classified. Training and classification were applied to all of the available ILC cohorts (G2 METABRIC *n* = 64, G2 RATHER *n* = 45, TCGA *n* = 81 and a G2 combined cohort *n* = 138).

### Survival analysis

The association of each gene with breast cancer-specific survival was assessed using the METABRIC ILC samples, first by a univariate approach and then also using a multivariate model. For the univariate analysis, the survival^71^ and rms^72^ R packages were used. Patients were split into quartiles based on expression level and survival curves plotted using the Kaplan–Meier method. The significance of differences between survival curves was evaluated using a log rank test, and a *P*-value of <0.01 was considered significant. The multivariate model was used to evaluate the prognostic ability of groups of genes in ILC tumors and compliment the survival analysis described above. A Cox proportional hazards model combined with a variable selection technique known as component-wise likelihood-based boosting^73^ was used to select a representative set of probes from the gene probes identified in the integrated analysis. The R package *CoxBoost*^73^ was used to implement the boosting algorithm, where the number of boosting steps was determined by 10-fold cross-validation. The genes from both the univariate survival analysis and multivariate CoxBoost analysis were evaluated and combined to form a gene expression signature (LobSig).

### Pathway analysis

Differentially expressed prognostic genes in ILC were analyzed using GeneGo Pathways Software (MetaCore; https://portal.genego.com/). Pathways were considered significant if there was *P*-value < 0.05 and visualized using REVIGO.^74^

### Reporting summary

Further information on research design is available in the Nature Research Reporting Summary linked to this article.

## Supplementary information

Supplementary Figures

Supplementary Tables

Reporting Summary

## Data Availability

The code for performing key analyses in this study is hosted at https://samirlal2.github.io/LobSig/.

## Supplementary information

**Supplementary Information** accompanies the paper on the *npj Breast Cancer* website (10.1038/s41523-019-0113-y).

## Data availability

The datasets generated during the current study is available in the GEO repository (accession GSE98528), or are included in this published article (and its supplementary information files). Additional datasets analyzed during this study are available from TCGA data portal (http://cancergenome.nih.gov/, data status as at May 15, 2014); *n* = 125 ILC from the METABRIC cohort (EGAS00000000083). Datasets that support Fig. 1–5, supplementary Figs. 2–7 and datasets supporting all supplementary tables are available upon request from the author as described at 10.6084/m9.figshare.8118743. The data generated and analyzed during this study are described in the following data record 10.6084/m9.figshare.8118743.^75^
