# Supplementary material for: LobSig is a multigene predictor of outcome in invasive lobular carcinoma
Source: NPJ Breast Cancer. 2019 Jun 27;5:18. doi: 10.1038/s41523-019-0113-y (PMC6597578; doi:10.1038/s41523-019-0113-y)
Supplement: Supplementary file 1 — Supplementary Figures [file 41523_2019_113_MOESM1_ESM.pdf]

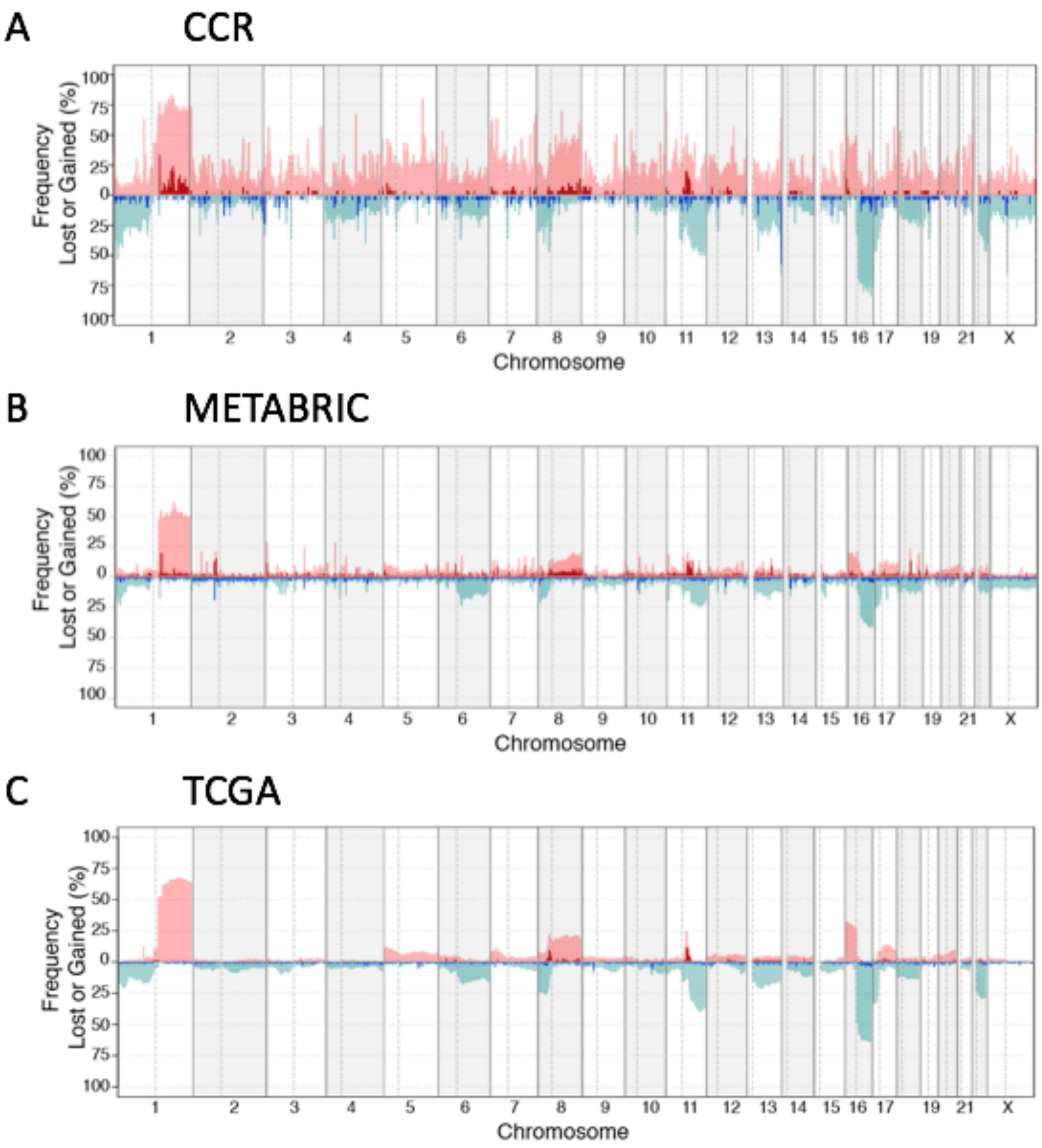

Supplementary Figure 1: Global recurrent alterations are conserved across different cohorts. Global recurrent alterations are plotted along the genome across different cohorts. Homozygous deletions (dark blue); copy number loss (light blue); copy number gain (pink); amplification (red). A) CCR cohort (n=30). B) METABRIC cohort (n=125). C) TCGA cohort (n=148).

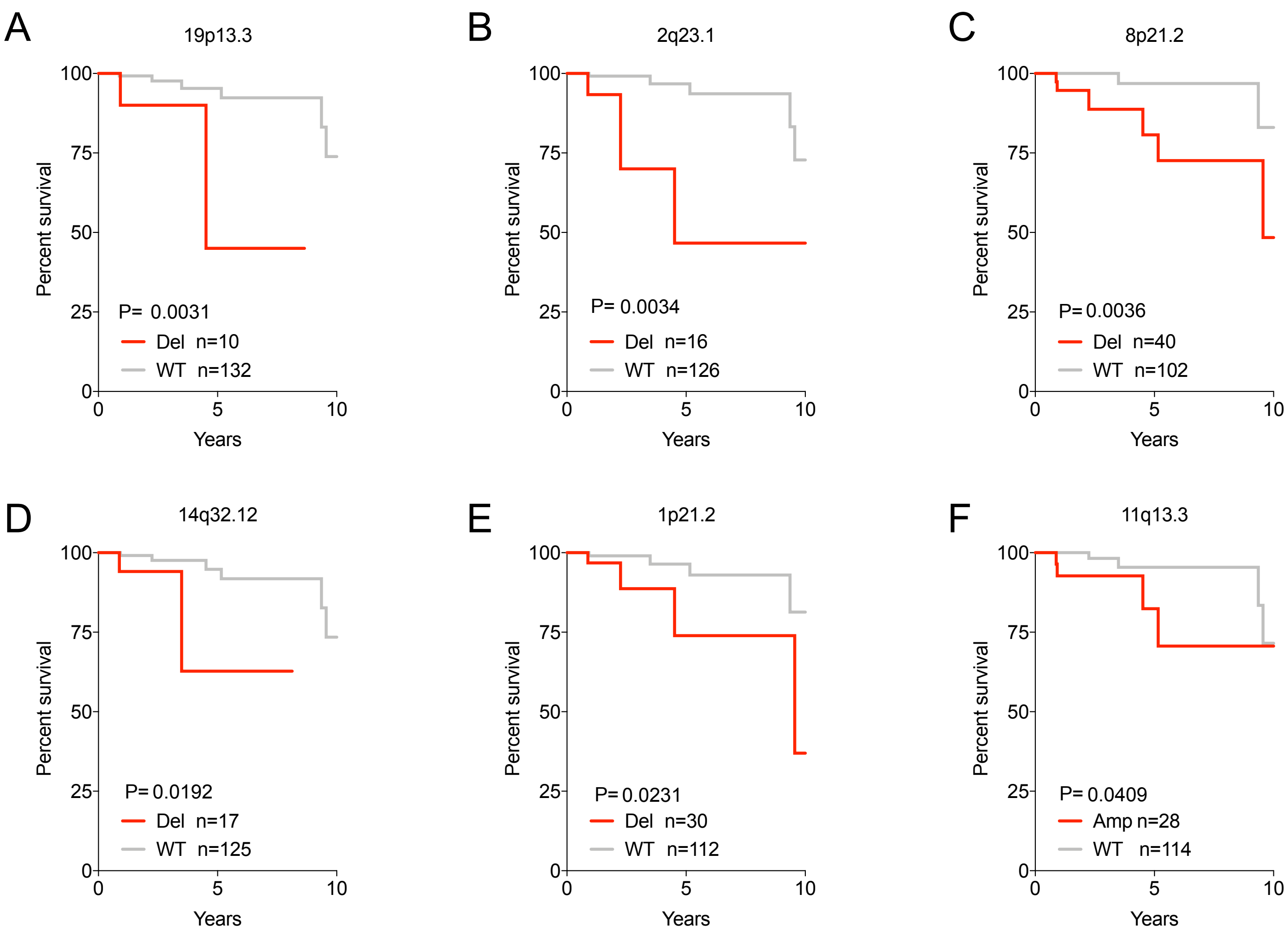

Supplementary Figure 2: Breast cancer specific survival is significantly associated with focal alterations as identified by GISTIC.
KM curves assessing survival outcomes for TCGA patients with the noted genomic alteration. A) 19p13.3, B) 2q23.1, C) 8p21.2, D) 14q32.12, E) 1p21.2, F) 11q13.3. Amp, amplification; Del, deletion; WT, wildtype copy number at the stated locus.

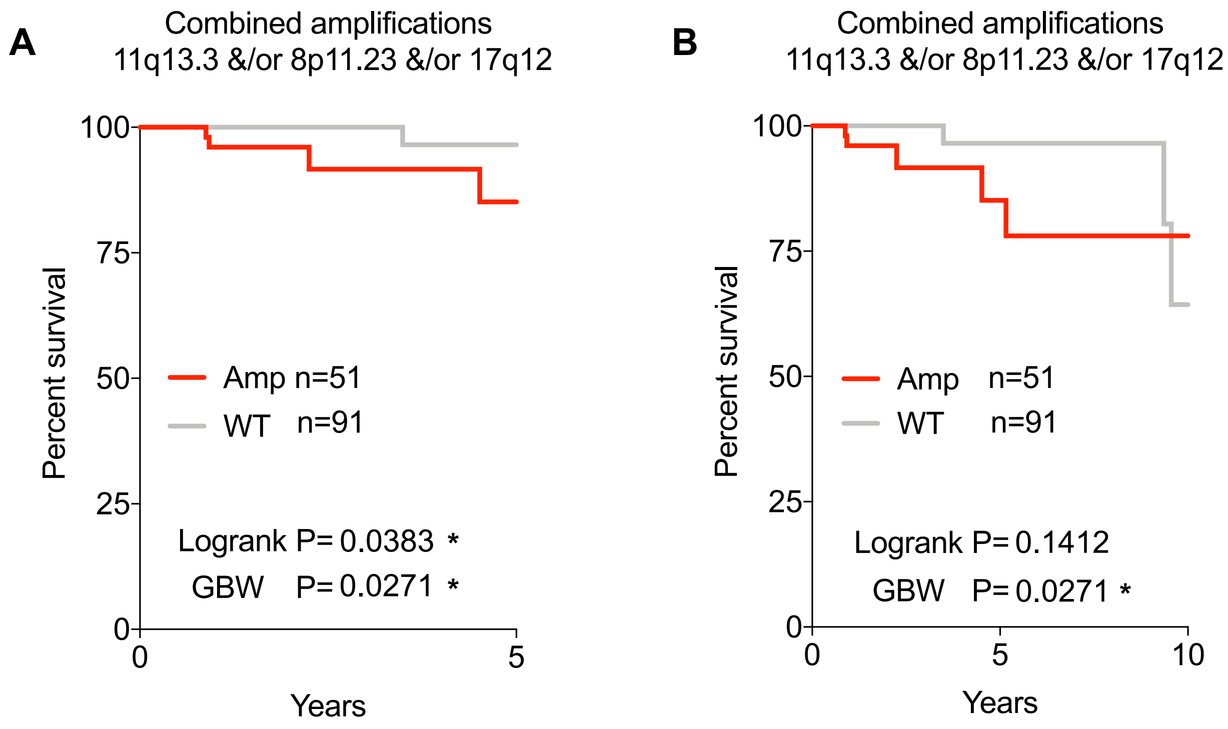

Supplementary Figure 3: Breast cancer specific survival is associated with focal co-amplifications as identified by GISTIC. KM curves depicting breast cancer specific survival for TCGA patients with amplifications at any of the following regions: 11q13.3, 8p11.23, 17q12. A) 5-year outcomes and B) 10-year outcomes, with significance indicated by *. Amp, amplification; GBW, Gehan-Breslow-Wilcoxon test; WT, wildtype copy number at the stated locus.

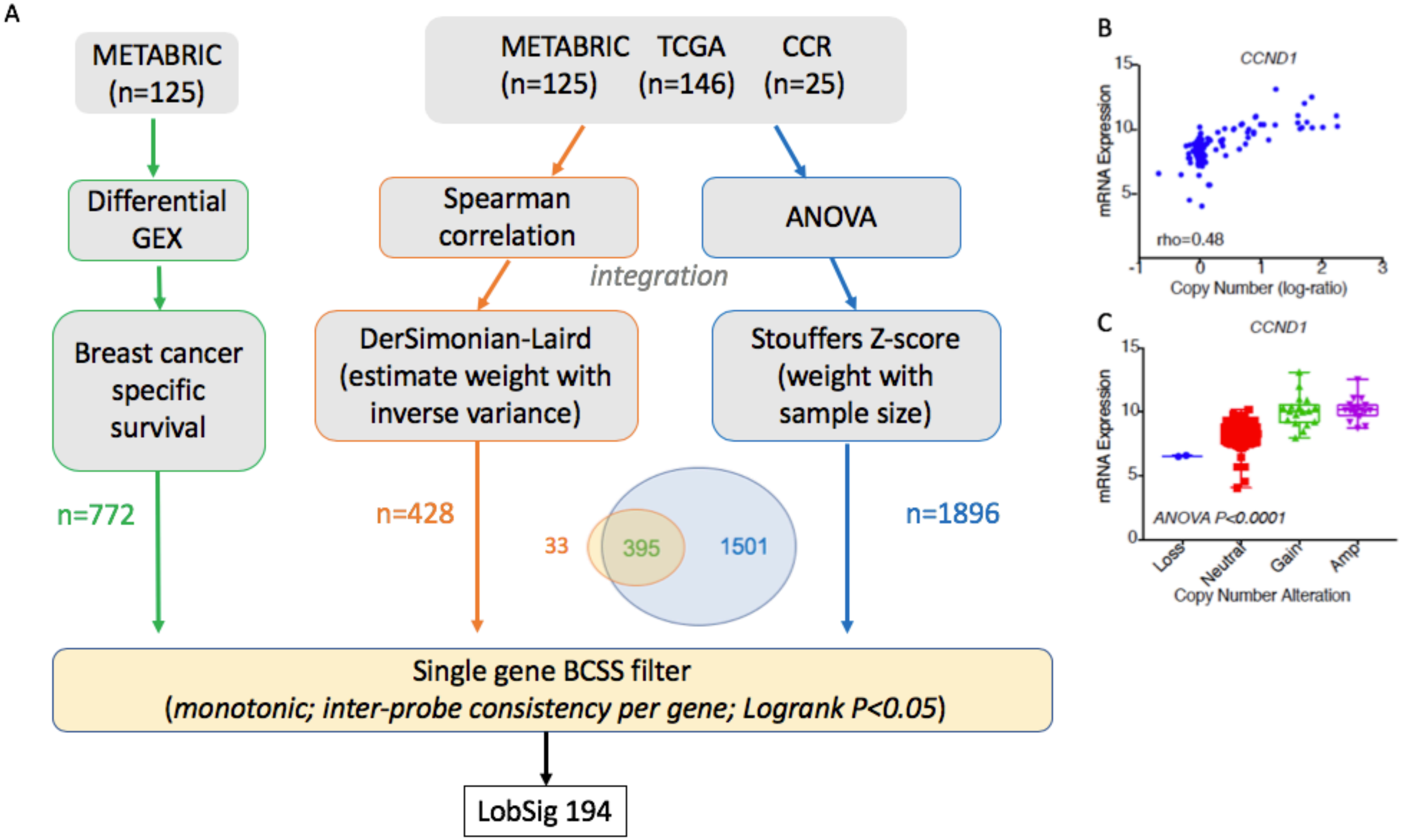

Supplementary Figure 4: Experimental design rationale. A) Flow chart of experiment design, including streams of differential GEX and integration. Cohorts were assembled from publicly available cohorts and our in-house cohort. Integration resulted in a Venn demonstrating that 395 genes were identified by both methods of data integration (*i.e.* gene expression levels were dictated by gene copy numbers changes). **B)** and **C)** demonstrate the necessity to perform a dual approach, as *CCND1* would not have been identfied by the Spearman analysis alone: B) Scatter plot showing the correlation of *CCND1* copy number state and gene expression data; the Spearman analysis. C) Boxplot showing the relationship between CCND1 gene expression and gene copy number state; ANOVA analysis.

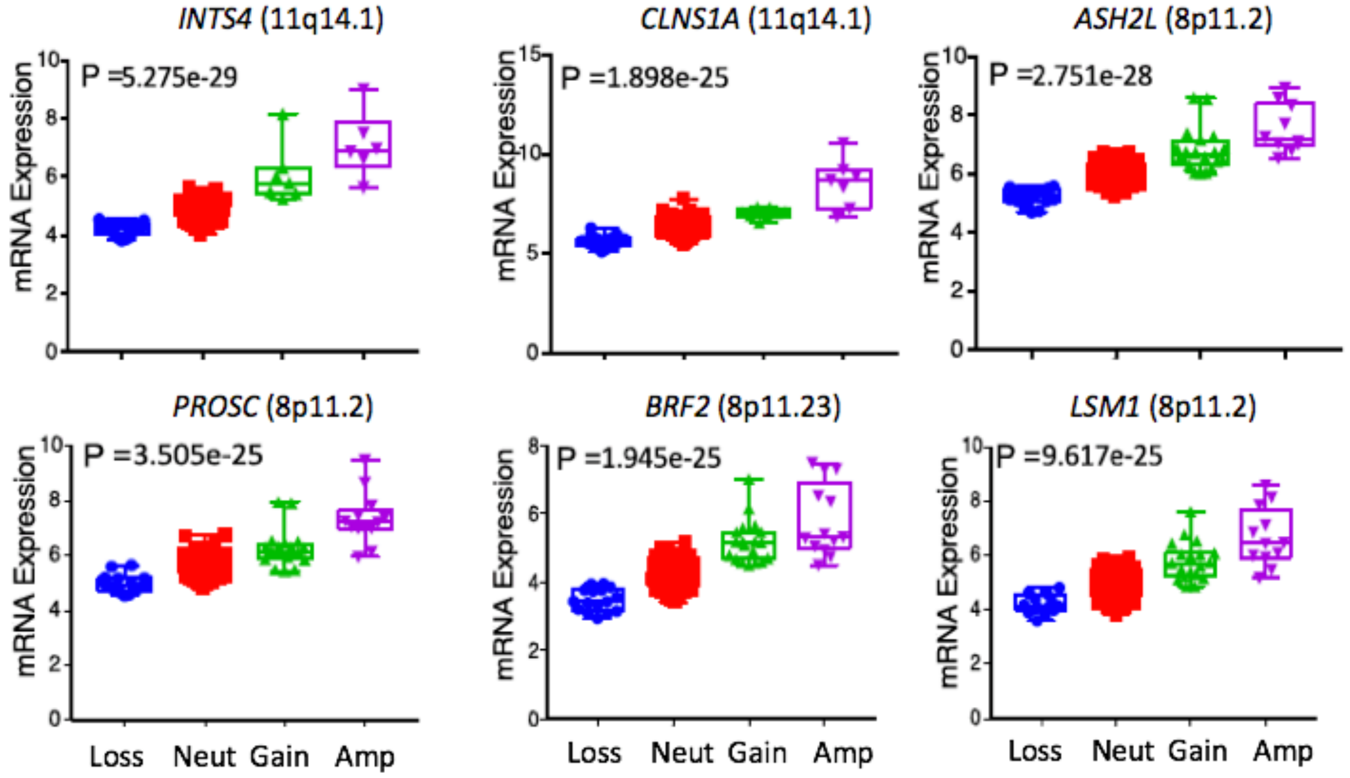

Supplementary Figure 5: Top six ANOVA genes

The x-axis of the scatter plot represents the copy number states (neut, copy alteration neutral) while the y-axis represents the gene expression values (voom normalized). The central line is the median, with whiskers extending from the 25th and 75th percentiles. The ANOVA p-values are as shown.

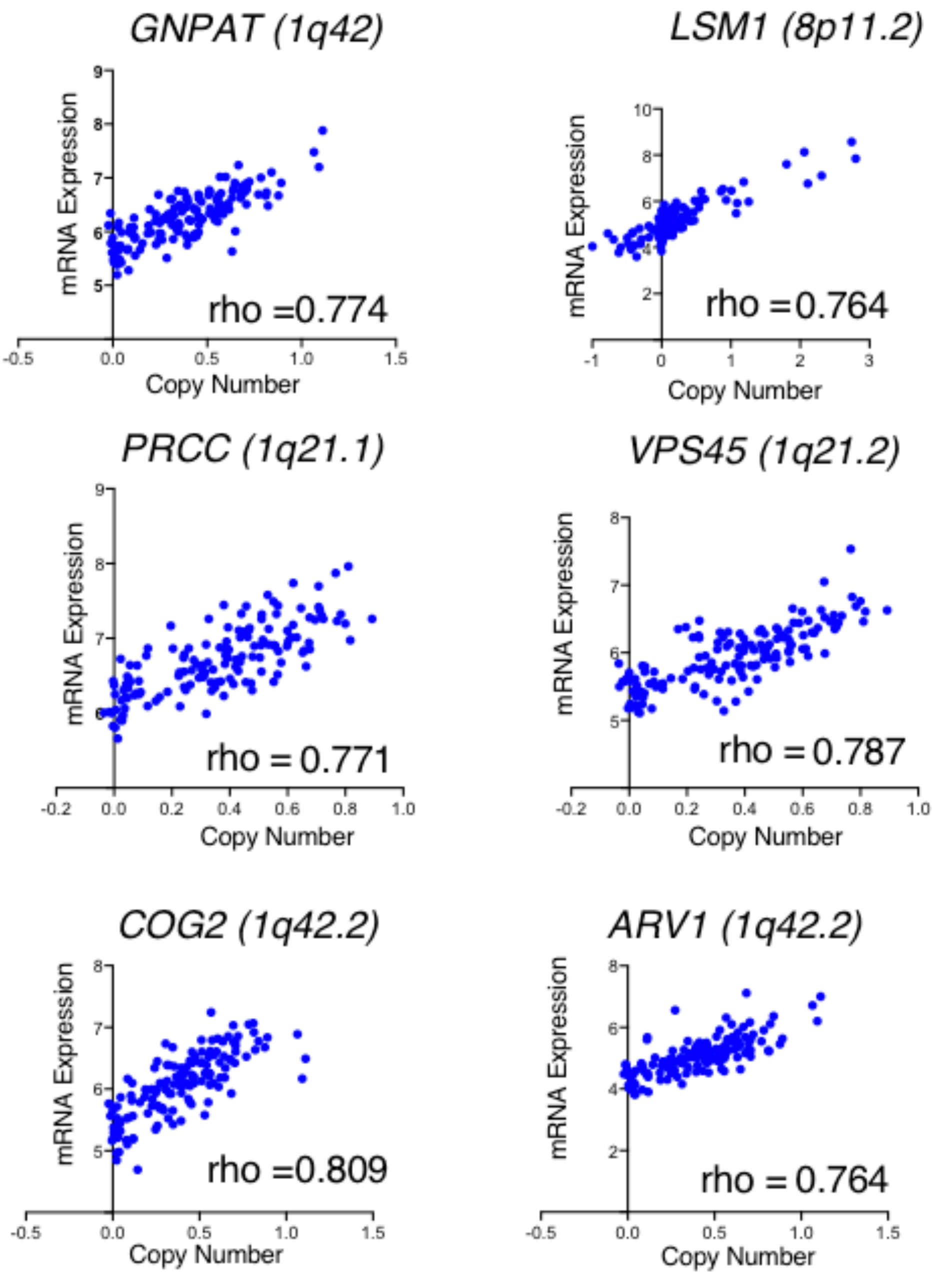

Supplementary Figure 6: Top six Spearman genes.

The x-axis of the scatter plot represents the continuous log2 CBS-smoothed copy number ratio values while the y-axis represents the mRNA expression as determined by RNA-Seq (voom normalized) from the TCGA cohort. The correlation coefficient values (rho) are as shown. The copy number values vary for each plot due to different frequencies of gain at 1q21.2 and 1q42.2 loci.

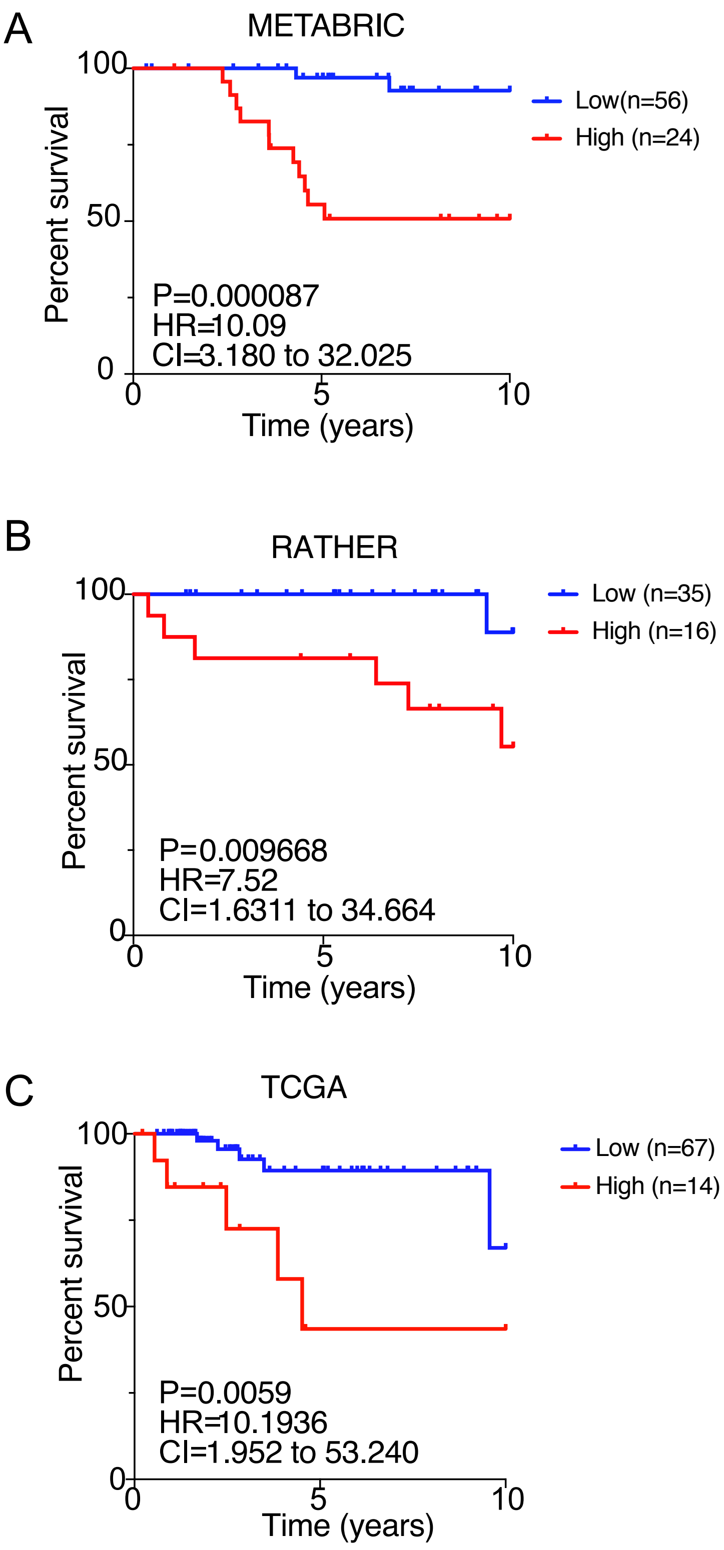

Supplementary Figure 7: Performance of LobSig model in independent cohorts. Kaplan-Meier plots of LobSig stratified groups in different cohorts A) METABRIC, B) RATHER and C) TCGA, with the Logrank P-value, hazard ratio and confidence intervals are reported below each curve.
